# Imaging characteristics of 4th ventricle subependymoma

**DOI:** 10.1007/s00234-022-02944-7

**Published:** 2022-04-15

**Authors:** Ali S. Haider, Tarek Y. El Ahmadieh, Maryam Haider, Kimmo J. Hatanpaa, Marco C. Pinho, Bruce E. Mickey, Raymond Sawaya, Gregory N. Fuller, Donald F. Schomer, Maria Gule-Monroe

**Affiliations:** 1 Department of Neurosurgery, The University of Texas MD Anderson Cancer Center, Houston, TX USA; 2 Department of Neurological Surgery, The University of Texas Southwestern Medical Center, Dallas, TX USA; 3 Department of Neuroradiology, The University of Texas MD Anderson Cancer Center, 1400 Pressler Street, Houston, TX 77030 USA; 4 Department of Pathology, The University of Texas Southwestern Medical Center, Dallas, TX USA; 5 Department of Radiology, The University of Texas Southwestern Medical Center, Dallas, TX USA; 6 Faculty of Medicine, American University of Beirut, Beirut, Lebanon; 7 Department of Pathology, The University of Texas MD Anderson Cancer Center, Houston, TX USA

**Keywords:** Subependymoma, Fourth ventricle, Neuroimaging, Rare tumors

## Abstract

**Purpose:**

Subependymomas located within the 4th ventricle are rare, and the literature describing imaging characteristics is sparse. Here, we describe the clinical and radiological characteristics of 29 patients with 4th ventricle subependymoma.

**Methods:**

This is a retrospective multi-center study performed after Institutional Review Board (IRB) approval. Patients diagnosed with suspected 4th ventricle subependymoma were identified. A review of clinical, radiology, and pathology reports along with magnetic resonance imaging (MRI) images was performed.

**Results:**

Twenty-nine patients, including 6 females, were identified. Eighteen patients underwent surgery with histopathological confirmation of subependymoma. The median age at diagnosis was 52 years. Median tumor volume for the operative cohort was 9.87 cm^3^, while for the non-operative cohort, it was 0.96 cm^3^. Thirteen patients in the operative group exhibited symptoms at diagnosis. For the total cohort, the majority of subependymomas (*n* = 22) were isointense on T1, hyperintense (*n* = 22) on T2, and enhanced (*n* = 24). All tumors were located just below the body of the 4th ventricle, terminating near the level of the obex. Fourteen cases demonstrated extension of tumor into foramen of Magendie or Luschka.

**Conclusion:**

To the best of our knowledge, this is the largest collection of 4th ventricular subependymomas with imaging findings reported to date. All patients in this cohort had tumors originating between the bottom of the body of the 4th ventricle and the obex. This uniform and specific site of origin aids with imaging diagnosis and may infer possible theories of origin.

## Introduction

Subependymoma is a rare central nervous system neoplasm that is classified as a grade I tumor by the World Health Organization (WHO) [1–4]. It is of subependymal origin and represents approximately 0.2–0.7% of all intracranial tumors [2, 5–7]. The first pathological description of subependymoma was reported in 1945, with few clinical studies reported since then [1, 8, 9]. These lesions are typically discovered incidentally and are usually asymptomatic [10].

Subependymomas are most commonly found in the fourth and lateral ventricles; however, a number of studies have reported lesions located in the spinal cord [3, 11–13]. Histologically, they appear as clusters of isomorphic nuclei embedded in a dense glial fibrillary matrix. Symptomatic lesions most frequently present with hydrocephalus and headache. Surgery is considered the primary treatment option for symptomatic cases [9, 14, 15]. There is a paucity in the literature regarding the imaging characteristics of these rare tumors. Here, we describe the clinical and radiological characteristics of 29 patients with fourth ventricle subependymoma as observed in two tertiary care academic medical centers.

## Methods

### Study design

This retrospective case series was performed after Institutional Review Board (IRB) approval at two participating institutions. Patients with intracranial subependymoma were identified via a database search at the University of Texas M.D. Anderson Cancer Center and the University of Texas Southwestern Medical Center. Patients 18 years of age or older diagnosed with fourth ventricle subependymoma via magnetic resonance imaging (MRI) or histopathology from 1993 to 2019 were included in the study.

Exclusion criteria consisted of patients younger than 18 years of age, subependymoma located outside the fourth ventricle, or absence of MRI images. A retrospective review of clinical, radiological (MRI), and pathological data was performed. MRI appearance was used to make the diagnosis of subependymoma in cases where surgical pathology was unavailable. All MRI images were reviewed by 3 fellowship-trained, board certified neuroradiologists with greater than 4 years of experience.

### Study variables

Patient demographic and imaging variables such as age, gender, date of initial MRI, date of last MRI, date of surgery, and symptoms at diagnosis were collected. Specific imaging variables for each patient collected included tumor location, tumor size, degree of enhancement, intensity of enhancement, appearance on T1, T2, FLAIR, DWI, GRE, and any change in imaging. The degree of enhancement was further characterized as non-enhancing, partially enhancing, and completely enhancing. The intensity of enhancement was further characterized based on the highest degree of enhancement into 3 categories: avid, moderate, and mild. Avid enhancement was defined as enhancement intensity similar to homogeneous dural venous sinus enhancement without flow artifacts. Moderate enhancement was defined as enhancement intensity similar to the degrees of enhancement of the choroid plexus. Mild enhancement was defined as enhancement intensity less than that of the choroid plexus. The change in imaging was defined as a minimum 25% increase in baseline tumor size for patients managed conservatively, while for patients treated via surgery, the change in imaging was defined as tumor recurrence.

### Statistical analysis

Tumor volume was calculated as [Anteroposterior × Transverse × Craniocaudal]. Analysis for descriptive statistics was performed using the statistical software SPSS V.24 (IBM Corp., Armonk, New York).

## Results

### Demographics and clinical features

Twenty-nine patients, including 6 females, were included. Eighteen patients (62%) underwent surgery with histopathological confirmation of subependymoma. The median age at diagnosis was 52 years. The median tumor volume for the operative cohort was 9.87 cm^3^, while for the non-operative cohort, it was 0.96 cm^3^. For the total cohort, the cranio-caudal diameter range was (min) 0.56 cm–(max) 5.60 cm and median was 2.00 cm. Thirteen patients (45%) were symptomatic on presentation, all of whom subsequently underwent surgical resection. The most common presenting symptoms were headache (*n* = 6) and dizziness (*n* = 5). The median length of follow-up in the operative cohort was 25.90 months, and no tumor recurrence was noted after surgical resection. In the non-operative cohort, the median length of follow-up was 65.70 months and all cases demonstrated no change in baseline tumor size (Table 1).

**Table 1:** Patient demographics and radiological characteristics

Patient demographics				
Total cohort		29 cases		
Non-operative cohort		11 cases		
Operative cohort		18 cases		
Gender		23 M; 6 F		
Age (median)		52 years		
Radiological characteristics				
		Total cohort	Operative	Non-operative
Tumor volume (cm^3^)		Median: 2.70	Median: 9.87	Median: 0.96
Craniocaudal diameter (cm)		Range (0.56–5.60) Median 2.00	Range (0.70–5.60) Median 2.25	Range (0.56–2.61) Median 1.65
T1 (*n* = 29)				
	Isointense	22/29 cases	14/18 cases	8/11 cases
	Hyperintense	1/29 cases	0/18 cases	1/11 cases
	Hypointense	4/29 cases	2/18 cases	2/11 cases
	Mixed (Iso + Hypo)	2/29 cases	2/18 cases	0/11 cases
T2 (*n* = 29)				
	Isointense	5/29 cases	2/18 cases	3/11 cases
	Hyperintense	22/29 cases	15/18 cases	7/11 cases
	Hypointense	1/29 cases	0/18 cases	1/11 cases
FLAIR (*n* = 28)				
	Hyperintense	28/28 cases	17/17 cases	11/11 cases
GRE/T2* (*n* = 20)				
	Susceptibility present	15/20 cases	9/10 cases	6/10 cases
DWI (*n* = 27)				
	Restricted diffusion	1/27 cases	1/17 cases	0/10 cases
Enhancement (*n* = 29)				
Present		24/29 cases	15/18 cases	9/11 cases
Location Centered below body of 4th ventricle		29/29 cases	18/18 cases	11/11 cases
Extension into Foramen of Magendie or Luschka		14/29 cases	13/18 cases	1/11 cases

*n* = number of patients that had this imaging sequence done

### MRI characteristics

For the total cohort, the majority of subependymomas (*n* = 22; 76%) were isointense on T1-weighted sequences and hyperintense (*n* = 22; 76%) on T2-weighted sequences. All subependymomas with available FLAIR imaging (*n* = 28; 97%) demonstrated FLAIR hyperintensity. Out of the 27 patients that had a DWI sequence available for review, only one subependymoma demonstrated restricted diffusion on diffusion weighted imaging. Susceptibility artifact of the tumor was present in 15 out of 20 patients with T2* imaging available for review. The susceptibility artifact correlated with coarse calcification on non-contrast CT of the head in 2 patients that had a CT available for comparison.

**Fig. 1:**
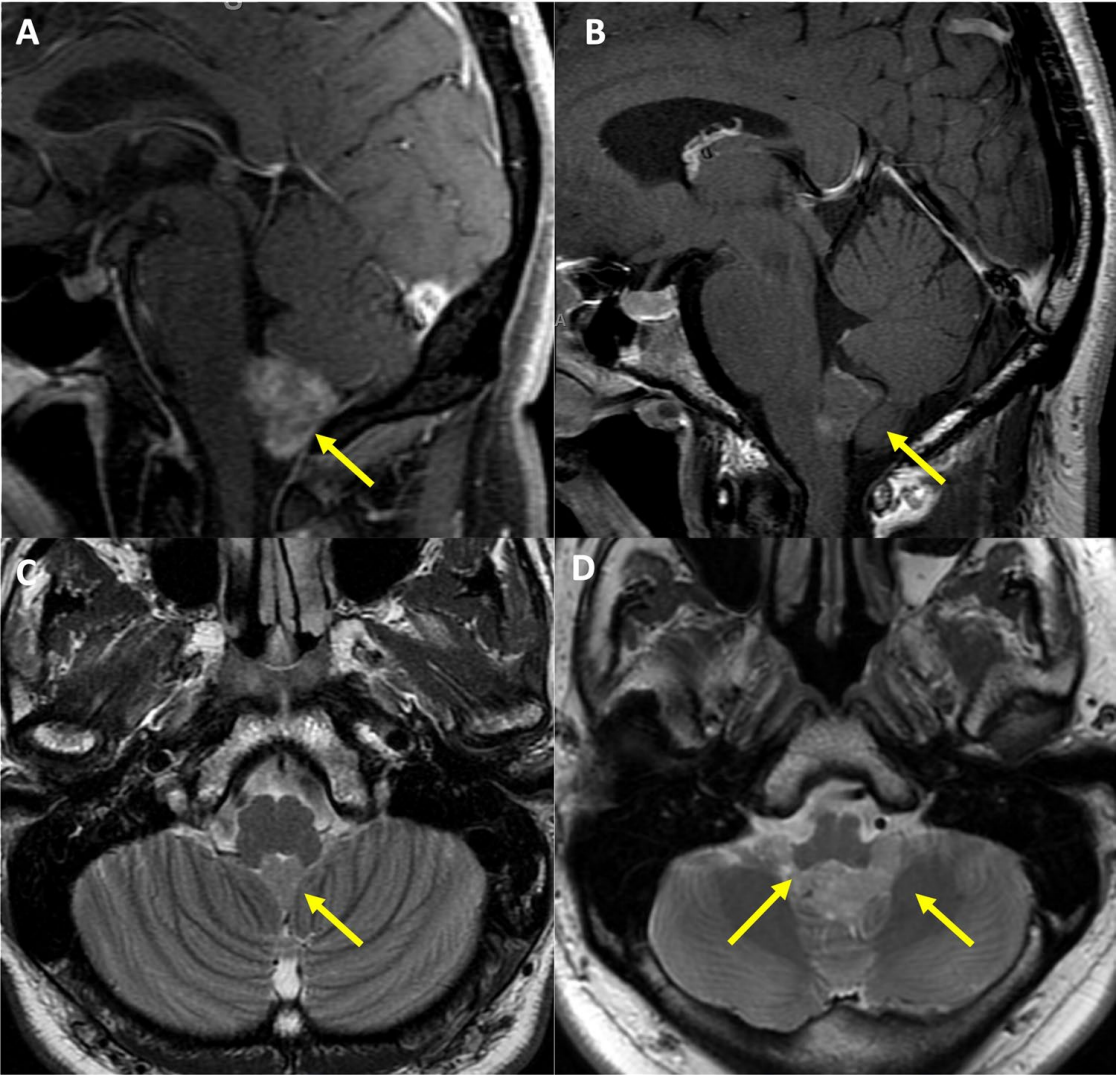
**A** Sagittal T1 contrast enhanced image demonstrates the presence of a moderately enhancing 4th ventricular mass below the body of the ventricle and extending into the obex. **B** Sagittal T1 contrast enhanced image demonstrates a minimally enhancing mass (predominantly T1 isointense) again situated in the low fourth ventricle, below the body of the fourth ventricle and illustrating the spectrum of enhancement seen with subependymomas. **C** Axial T2 image of a typical mildly T2 hyperintense subependymoma at midline extending toward the foramen of Magendie. **D** Axial T2 image of a more T2 hyperintense subependymoma extending through the foramen of Luschka bilaterally

**Fig. 2:**
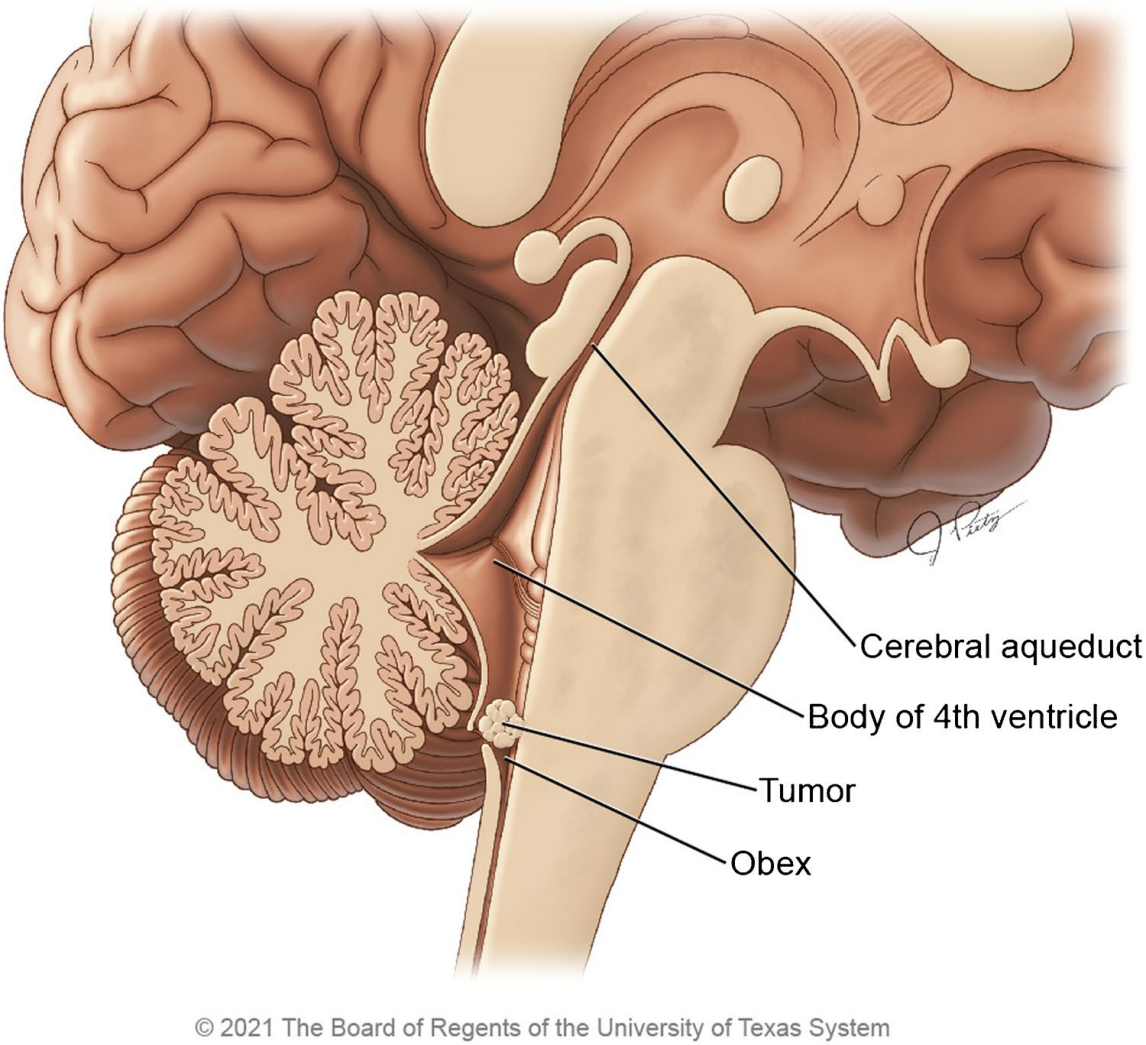
Sagittal illustration demonstrating a subependymoma centered below the body of the 4^th^ ventricle and terminating near the level of the obex

The majority of subependymomas were enhanced on post-contrast T1-weighted sequence (*n* = 24; 83%). However, enhancement was only partial, with no tumors found to be completely enhancing. The intensity of enhancement ranged from mild (*n* = 6; 21%) to moderate (*n* = 16; 55%), along with one tumor demonstrating avid enhancement and one tumor considered too heterogeneous in intensity to further classify in either category (Fig. 1A–D). Within the 4th ventricle, all tumors (*n* = 29; 100%) were noted to be centered below the body of the 4th ventricle and terminating near the level of the obex. Three patients had obstructive hydrocephalus at the time of presentation. Fourteen cases (48%), 13 of which were in the operative cohort, demonstrated tumor extension into the foramen of Magendie or Luschka (Fig. 2). Median tumor volume for cases with foramen extension was 10.95 cm^3^, while for the cases without foramen extension, it was 1.13 cm^3^.

## Discussion

In this study, we report the largest imaging experience to date on 4th ventricular subependymoma. The majority of patients in this study were confirmed histopathologically as subependymomas. Also included were adult patients with tumors of the 4th ventricle that exhibited imaging stability over time in keeping with low-grade neoplasm, thus excluding other known pathologic entities of the 4th ventricle known to be progressive lesions such as ependymoma or medulloblastoma [21–25]. This strategy also excluded 4th ventricular tumors that although low-grade are known to be symptomatic requiring surgery such as choroid plexus papillomas or pilocytic astrocytoma and known to have an aggressive course when presenting in adulthood [22–28]. Furthermore, the MR imaging characteristics appeared relatively uniform between the operative and non-operative cohort, supporting a common pathology.

Our study is the first to identify a specific site of origin for 4th ventricular subependymomas. All patients in our cohort were found to have tumors originating below the body of the 4th ventricle and above the obex. This observation in conjunction with long-term imaging stability suggests the likely diagnosis for these rare tumors. Furthermore, the presence of tumor at the foramen of Luschka or Magendie as well as larger tumor size was observed to be linked with clinical symptoms requiring surgical resection. This is in contrast to the patients that were placed on imaging surveillance where no patients exhibited symptoms, only one patient had tumor at the foramen of Luschka, and tumors were noted to be smaller in size compared to the operative cohort.

As noted in the literature, intracranial subependymomas are most commonly found in the fourth and lateral ventricles [10, 16–18]. Within the fourth ventricle, we observed all of the subependymomas in our study to be centered below the body of the fourth ventricle and terminating at the level of the obex. We postulate that this neuroanatomical finding may have a developmental relationship with the area postrema, a circumventricular paired structure in the medulla oblongata of the brainstem [19]. The area postrema is located at the floor of the fourth ventricle, highly vascular, and lined by glial cells. The cell of origin for subependymomas is thought to originate from neural stem cells. Bennett et al. have suggested that circumventricular organs such as the area postrema may be a novel source of neural stem cells, raising the developmental concept of subependymomas originating in this area [20]. Obviously, this hypothesis requires further investigation.

Radiographic findings in our study revealed that fourth ventricle subependymomas are more commonly isointense on T1, hyperintense on T2, enhance, and demonstrate FLAIR hyperintensity. The presence of susceptibility artifact was seen in 75% of our patients and was found to represent coarse calcification on CT in a limited number of patients that had CT available for comparison. Though the majority of cases in our study demonstrated enhancement, it is worth noting that most of these tumors exhibited relatively mild to moderate enhancement. This is generally consistent with what has been previously described in multiple retrospective case series with regards to intracranial subependymoma [8, 10–12, 16–18, 21, 22]. It is worth noting that none of our lesions demonstrated complete enhancement, and only one demonstrated avid enhancement. This may be helpful when differentiating from lesions with characteristic complete or avid enhancement, such as papillomas, meningiomas, and hemangioblastomas [23–28].

Nearly half of the cases in our study demonstrated tumor extension outside of the fourth ventricle, either into foramen of Magendie or Luschka. Though sparingly noted in the literature, no studies have drawn any correlation between subependymoma extension outside of the fourth ventricle and diagnosis [18]. However, we observed that the larger tumors in our study appeared to have a greater incidence of extension outside of the fourth ventricle.

There was an appreciable difference in tumor size between the operative and non-operative cohorts in our series, with the operative tumor cohort being consistently larger in size than the non-operative cohort. All symptomatic lesions in our study underwent surgical resection. The most common symptoms noted in our study included headache and dizziness, which are consistent with the literature [10, 14, 22, 29]. Obstructive hydrocephalus was also observed in three of our patients before surgery, which was noted in the literature to be a particular sequelae of fourth ventricle subependymoma [10, 17, 18]. None of the cases in our study experienced tumor recurrence, consistent with the literature regarding the clinical management and cellular growth of intracranial subependymoma [8, 9, 12–15, 30].

Our study is a retrospective case series pooling data from two large tertiary academic medical centers. It has several limitations, including inherent bias due to the retrospective and observational nature of the study. A possible geographic bias regarding patient demographics can also limit our study due to both participating institutions being in the same region, while patient insurance status can also possibly bias the patient cohort that was available for our study. Our small sample size may be biased toward larger or more symptomatic lesions, since asymptomatic lesions are less likely to present and undergo imaging. Also, our cohort consists of only 18 patients with a histologically confirmed diagnosis. Our length of follow-up is relatively short; however, it is understood that these are benign tumors. Furthermore, a larger study would be difficult to perform for such rare and indolent tumors.

## Conclusions

To the best of our knowledge, this study is the largest imaging experience of 4th ventricular subependymomas reported in the literature to date. These tumors are noted to have characteristic imaging features and a propensity to be located below the body of the fourth ventricle, terminating at the level of the obex. Larger, prospective multi-center studies are required to further determine the clinical and imaging characteristics of these tumors.

## Data Availability

The datasets generated/analyzed during this study are not publicly available due to data protection but are available from the corresponding author upon reasonable request.

## Code availability

N/A.

## References

[1] Scheinker I (1945). Subependymoma: a newly recognized tumor of subependymal derivation. J Neurosurg.

[2] Louis DN (2016). The 2016 World Health Organization classification of tumors of the central nervous system: a summary. Acta Neuropathol.

[3] Scheithauer BW (1978). Symptomatic subependymoma. Report of 21 cases with review of the literature. J Neurosurg.

[4] Louis DN (2021). The 2021 WHO Classification of Tumors of the central nervous system: a summary. Neuro Oncol.

[5] Moss TH (1984). Observations on the nature of subependymoma: an electron microscopic study. Neuropathol Appl Neurobiol.

[6] Boykin FC (1954). Subependymal glomerate astrocytomas. J Neuropathol Exp Neurol.

[7] Godwin JT (1959). Subependymal glomerate astrocytoma; report of two cases. J Neurosurg.

[8] Bi Z (2015). Clinical, radiological, and pathological features in 43 cases of intracranial subependymoma. J Neurosurg.

[9] Rushing EJ (2007). Subependymoma revisited: clinicopathological evaluation of 83 cases. J Neurooncol.

[10] Jain A (2012). Subependymoma: clinical features and surgical outcomes. Neurol Res.

[11] Chiechi MV, Smirniotopoulos JG, Jones RV (1995). Intracranial subependymomas: CT and MR imaging features in 24 cases. AJR Am J Roentgenol.

[12] D'Amico RS (2017). Subependymomas are low-grade heterogeneous glial neoplasms defined by subventricular zone lineage markers. World Neurosurg.

[13] Lombardi D (1991). Symptomatic subependymoma: a clinicopathological and flow cytometric study. J Neurosurg.

[14] Nguyen HS (2017). Intracranial subependymoma: a SEER analysis 2004–2013. World Neurosurg.

[15] Ragel BT (2006). Subependymomas: an analysis of clinical and imaging features. Neurosurgery.

[16] Hoeffel C (1995). MR manifestations of subependymomas. AJNR Am J Neuroradiol.

[17] Jooma R (1985). Subependymomas of the fourth ventricle. Surgical treatment in 12 cases. J Neurosurg.

[18] Kammerer S (2018). Subependymomas - characteristics of a "Leave me Alone" Lesion. Rofo.

[19] Mirza M, Das JM (2020). Neuroanatomy, Area Postrema.

[20] Bennett L (2009). Circumventricular organs: a novel site of neural stem cells in the adult brain. Mol Cell Neurosci.

[21] Ernestus RI, Schroder R (1993). Clinical aspects and pathology of intracranial subependymoma–18 personal cases and review of the literature. Neurochirurgia (Stuttg).

[22] Fujisawa H, Hasegawa M, Ueno M (2010). Clinical features and management of five patients with supratentorial subependymoma. J Clin Neurosci.

[23] Safaee M (2013). Choroid plexus papillomas: advances in molecular biology and understanding of tumorigenesis. Neuro Oncol.

[24] Zhang TJ (2011). MRI findings of choroid plexus tumors in the cerebellum. Clin Imaging.

[25] Elster AD (1989). Meningiomas: MR and histopathologic features. Radiology.

[26] Tamrazi B, Shiroishi MS, Liu CS (2016). Advanced Imaging of Intracranial Meningiomas. Neurosurg Clin N Am.

[27] Glasker S, Van Velthoven V (2005). Risk of hemorrhage in hemangioblastomas of the central nervous system. Neurosurgery.

[28] Slater A, Moore NR, Huson SM (2003). The natural history of cerebellar hemangioblastomas in von Hippel-Lindau disease. AJNR Am J Neuroradiol.

[29] Varma A (2018). Surgical management and long-term outcome of intracranial subependymoma. Acta Neurochir (Wien).

[30] Kong LY (2014). Therapeutic targets in subependymoma. J Neuroimmunol.

